# Current concepts and an alternative perspective on periodontal disease

**DOI:** 10.1186/s12903-020-01221-4

**Published:** 2020-08-26

**Authors:** Gunnar Dahlen, Ole Fejerskov, Firoze Manji

**Affiliations:** 1 Department of Oral Microbiology and Immunology, Institute of Odontology, Sahlgrenska Academy, University of Gothenburg, Box 450, 40530 Gothenburg, Sweden; 2 Department of Biomedicine, Faculty of Health, Aarhus University, Aarhus, Denmark; 3 Institute of African Studies, Carleton University, Ottawa, Canada

**Keywords:** Periodontitis, Oral microbiome, Inflammation, Host-pathogen response, Epidemiology

## Abstract

**Background:**

Epidemiological data from countries worldwide show a consistent pattern implying that a fraction of around 10% of those over 40–50 years in all populations will exhibit severe periodontitis with the potential risk of losing teeth during their life-time. The subgingival microbiota shows striking similarities between populations irrespective of disease severity and can only marginally explain the clinical pattern. It is also difficult to explain this pattern by genetic and acquired risk factors such as systemic disease (e.g. diabetes) or habits (e.g. smoking) even if they may have a confounding effect on the disease.

**Main text:**

Inflammation of the gingiva appears to be a normal and physiological response to the presence of commensal bacteria along the gingival crevice and in the dental biofilm. Over many years of exposure to the dental biofilm, the chronic inflammation in the gingiva gradually results in a loss of attachment and bone loss. Numerous laboratory and clinical studies have provided insight into the potential role of determinants that are associated with periodontitis. However, it has been difficult to relate the findings to the pattern of the distribution of the disease observed in epidemiological studies. We propose a simple and parsimonious model that considers all the multitude of potential determinants as creating effectively random noise within the dental biofilm to which the tissues react by accumulating the effects of this noise.

**Conclusions:**

We suggest that such a model can explain many of the epidemiological features of periodontal breakdown over time, and we discuss its clinical implications.

## Introduction

One of the most striking, and perhaps enigmatic, features of the epidemiology of periodontitis is the similarity in the patterns of periodontal loss of attachment in different populations across the world, whether or not they exhibit poor oral hygiene, or receive regular oral health care [1, 2]. What we find is that gingival inflammation of some degree is ubiquitous from childhood to old age; the progress of periodontitis is slow, with loss of attachment occurring after the age of 30–40 years, with some degree of loss occurring in everyone; advanced loss of attachment occurs in a minority of the population and increases with age to a prevalence of 10–15%; loss of attachment occurs on buccal and lingual surfaces often accompanied by gingival recession, whereas pocket formation predominates in proximal spaces, often bilaterally; and the core oral microbiome (commensals), including putative periodontopathogens, are widespread with a high carriage rate in human adults globally but appear to have a limited relationship with periodontal disease prevalence or severity.

In this paper we review the different theories or hypotheses proposed to explain these epidemiological features and suggest that while the many laboratory and clinical studies have thrown important light on our understanding of the many and complex determinants involved in periodontitis, they do not necessarily explain why the epidemiological features of periodontitis should be so universal. We explore how a simple random effects model that takes into account the effect on the tissues of a multitude of determinants associated with periodontitis and which might provide some understanding of why the features of periodontitis should be expected to occur universally.

### Periodontal disease

Periodontal disease is generally considered to be an inflammatory disease induced and maintained by the microbiota of the dental biofilm (dental plaque). The origin of this concept stems from the “experimental gingivitis in man” studies carried out by Löe and co-workers in the mid 60s [3]. The inflammatory response in the gingiva was thought to be the initial stage of a disease process, which over time was transformed into a destructive phase, periodontitis, characterised by loss of attachment and bone loss. This was the essence of the “non-specific plaque hypothesis” [4].

The role of microorganisms in periodontitis is, however, unclear although certain “pathogens” alone or in clusters have been proposed to play a major role [5–7]. This approach termed “the specific plaque hypothesis” dominated the periodontal microbiology for several decades. Antibiotics were proposed as the mode of treatment.

The “ecological plaque hypothesis” was introduced [8, 9], together with an expanded list of potential periodontopathogens [10], suggesting that the key factor in the disease process was the ecological shift to a dysbiosis. Prevention and treatment were focused on ways to prevent dysbiosis occurring [11–13].

Recently the key-stone hypothesis and the polymicrobial and dysbiosis hypothesis have been described in order to emphasize the interaction between the polymicrobial community and a dysregulated inflammatory response [14, 15]. Although detailed knowledge of the microbiome and its function has increased, the specific role of the microorganisms in periodontitis development and progression remains unclear.

Others have focused more on the quality of the inflammatory response and the influence of genetic and/or environmental factors (smoking, systemic diseases) [16, 17] to explain why certain individuals seem to be more “susceptible” and the research focus has turned increasingly towards the host response and systemic host effects rather than the role of the microbiota in the disease process [18]. Further, a gradual immune senescence by age and/or aging itself have been proposed to impact on oral health including periodontal disease [19, 20], while others have argued that inflammatory reactions and immune response in general both quantitatively and qualitatively may be genetically determined [21]. Consequently, gingivitis and periodontitis may be explained along genetic lines, although the evidence so far has limited predictive value and does not account for the apparent universal pattern observed in epidemiological studies.

While there are many hypotheses about the aetiology of periodontitis, the challenge has been to assess the extent to which they explain the epidemiological features of the disease [22].

A random “disease” model for periodontal destruction was launched as the “burst theory” already by Socransky et al. [23] based on a previous observation [24] that periodontal disease was a dynamic condition of disease exacerbation and remission as well as periods of inactivity. This was further dealt with and theoretically explained by Manji and Nagelkerke [25] how burst and remissions can occur as a direct consequence of the accumulation of random events. Unfortunately, this concept of explaining the periodontal disease has been neglected during the last 30 years in favor of the deterministic approach and search for “risk factors” for disease development. This approach has been questioned recently [26].

### Periodontal disease epidemiology

Pilot [27] concluded in his review that “from a public health perspective the relative similarities in periodontal conditions around the world are far more striking than the differences.” Subsequently, Kassebaum et al. [2] reported in a systematic review and meta-regression paper that severe periodontitis affected about 10.8% of the global adult population. The analyses indicated that prevalence increases dramatically between 35 and 44 years of age and with an incidence peak at 38 years of age. Despite the diversity of case-definitions, the diversity in the number of uncontrolled factors, as well as diverse methodologies employed in these studies, the prevalence of severe periodontitis shows a remarkable similarity [2, 28] . Similar conclusions were drawn from studies on periodontal epidemiology [1] indicating that the prevalence and extent of attachment loss increases with age in all populations and that the extent and severity of destruction tends to be skewed to such a degree that a small fraction of the subjects account for most of the destruction. This between population similarity of periodontal breakdown has been emphasized in a number of studies on natural disease development in populations with poor oral health and with little or no access to dental treatment in Sri Lanka [29], Tanzania [30], Kenya [31] China [32–34], Southern Thailand [35], and Northern Thailand [36]. In a comparative evaluation of the profiles of destructive periodontal disease in different populations with close to 100% presence of gingival bleeding, calculus and plaque [34], it was concluded that while the periodontal loss profiles may differ in severity or extent between populations, these differences do not conform with the traditional generalization that African and Asian populations suffer more severe periodontal breakdown than other populations. Already in the 1980s. Cutress et al. [37] suggested that the amount of plaque, calculus and gingival bleeding are of limited value for risk assessment of severe periodontal breakdown.

A similar pattern of loss of attachment has also been observed in Western Europe and North America populations where access to dental treatment is more widespread. In USA, a prevalence of severe periodontitis in adults > 30 years of age was found to be 8.9% [38]. In Sweden, it was reported a frequency of severe periodontitis to be 7% in adults 50 years of age [39].

The skewed distribution of attachment loss has been interpreted as indicative of the presence of ‘risk groups’ [40, 41], but it remains unclear why diverse populations across the world should exhibit similar patterns of breakdown or why there should be similar proportions of people that constitute ‘risk groups’.

Longitudinal studies on periodontal progression in populations with poor oral hygiene and no dental care are rare [33, 42]. In a 10-year study of the progression of destructive periodontal disease in adult and elderly Chinese, Baelum et al. [42] concluded that virtually all individuals will experience some attachment loss, while the distribution of attachment loss over the 10 year period was skewed in all age groups. The longitudinal data provided results not dissimilar to those found in cross-sectional studies. Baelum et al. [42] interpreted the findings in adult and elderly Chinese as suggesting that the “causes” of destructive periodontal disease are to be found in the host response to years of dental plaque exposure rather than factors related to the biofilm itself.

### Epidemiology of oral microbiota

Microbiological assessments in populations world-wide have shown a high prevalence of putative periodontal pathogens without being directly related to a periodontal disease prevalence [43]**.** Using culture methodologies, Dahlen et al. recorded a prevalence of *Porphyromonas gingivalis* in subgingival plaque samples in 70% of adult Kenyans and in more than 50% of adult Chinese [44, 45]. *Prevotella intermedia* was found in close to 100% of people examined in both populations (Table 1). Using Checkerboard methodology, a high prevalence (87.2–100%) was recorded for 27 different species among adults in rural Southern Thailand [46], and 83–100% for seven putative periodontopathogens in an adult Chines population [47]. Similar prevalences using checkerboard methods were found in adults in a remote population of Northern Thailand [36]. Further, a prevalence of 87% for *P. gingivalis*, 100% for *P. intermedia* and motile rods, 89% for spirochetes using an indirect immunofluorescens assay was found in individuals 15–25 years of age in Indonesia [48]. Using the same method, Preus et al. [49] found in young adults (15–25 years of age) in Sri Lanka somewhat lower prevalence of *P. gingivalis* (40%) and *P. intermedia* (76%).

**Table 1:** Prevalence (%) of “Potential periodontal pathogens” in some studies on adult populations with poor oral hygiene and limited access to dental care

Study	Country/ population	No of subjects	Age	Method of detection	Aa	Pg	Td	Tf	Pi	Fn	Cr
Dahlen et al. [44]	Kenya	20	30–65	Culture	40	70	Nd	Nd	100	Nd	Nd
Dahlen et al. [45]	China	60	55–69	Culture	10	50	Nd	Nd	93	Nd	80
Papapanou et al. [46]	China	70 78	30–39 50–59	CKB	83	100	97	98	100	100	100
Papapanou et al. [47]	Southern Thailand	207 149	30–39 50–59	CKB	97	99	99	99	99	99	98
Preus et al. [49]	Sri Lanka	268 males	15–30+	IF	15	40	Nd	Nd	76	Nd	Nd
Timmerman et al. [48]	Indonesia	255	15–25	IF	57 25	89 61	89 47	Nd Nd	100 99	Nd Nd	100^a^ 92
Kvarnvik et al. [36]	Northern Thailand	86	30–60	CKB	83	99	90	92	96	79	96

Abbrevatíons: *Nd* Not detected; *CKB* Checkerboard; *IF* Immunofluorescens; *Aa Aggregatibacter actinomycetemcomitans; Pg Porphyromonas gingivalis*; *Td Treponema denticola; Tf Tannerella forsythia*; *Pi Prevotella intermedia*; *Fn Fusobacterium nucleatum*; *Cr Campylobacter rectus*.

^a^indicate prevalence of motile rods

The similarities in the periodontal microbiota across populations in diverse geographical locations are striking. Gram-negative anaerobes and motile bacteria appear to predominate in gingival and periodontal pockets, and could be considered normal commensals. The proportions and load of various genera or species may vary between populations as well as between individuals within the population and sites (e.g. in deep pockets) within the same mouth, but the overall pattern of the oral core microbiota (microbiome) appears to be the same. While a dysbiosis may indeed occur [11, 14] this may not be a sufficient factor to explain the epidemiological features of periodontitis. It is possible that certain microorganisms have a stronger impact on the disease progression but the precise role of bacteria and “putative pathogens” in periodontitis still remains unclear [50].

### The microbiome and ecology of the gingival pocket (crevice)

The oral microbiome is different from the microbiome found in other body compartments such as skin, intestine and vagina [51]. The oral microbiome comprises a highly diverse microbial population, involving more than 700 species [52]. The dental biofilm has its own microbiome characterized by strong tooth-surface adhering streptococci and Actinomyces [46]. The dental biofilm appears to be in a dynamic state and self-regulating through the constant competition between the microorganisms for space, ecological conditions and nutrition. Since the streptococci and others of the *Firmicutes* phylum (*Granulicatella, Gemella, Veillonella*) have the capacity to degrade glycoproteins, they constitute the core microbiome of the dental plaque [53, 54]. The microenvironment along the gingival crevice is different from other parts of the tooth surface, the primary source of nutrition coming from gingival crevicular fluid (GCF), the quantity of which correlates with the degree of inflammation. Thus, it seems that the main source of nutrition for the microbiota in this niche is proteins and the main metabolic pathway is proteolytic, favouring the proteolytic rather than the saccharolytic microorganisms. In addition, the GCF, which is a serum exudate, also contains a number of growth supporting factors such as vitamins (K-vitamin or menadione), hormones (oestrogen) and specific serum proteins/peptides (hemin) all favouring many of the fastidious Gram-negative anaerobes that adapt and grow concomitantly with gingival inflammation and deepening gingival pockets [12, 54].

**Fig. 1:**
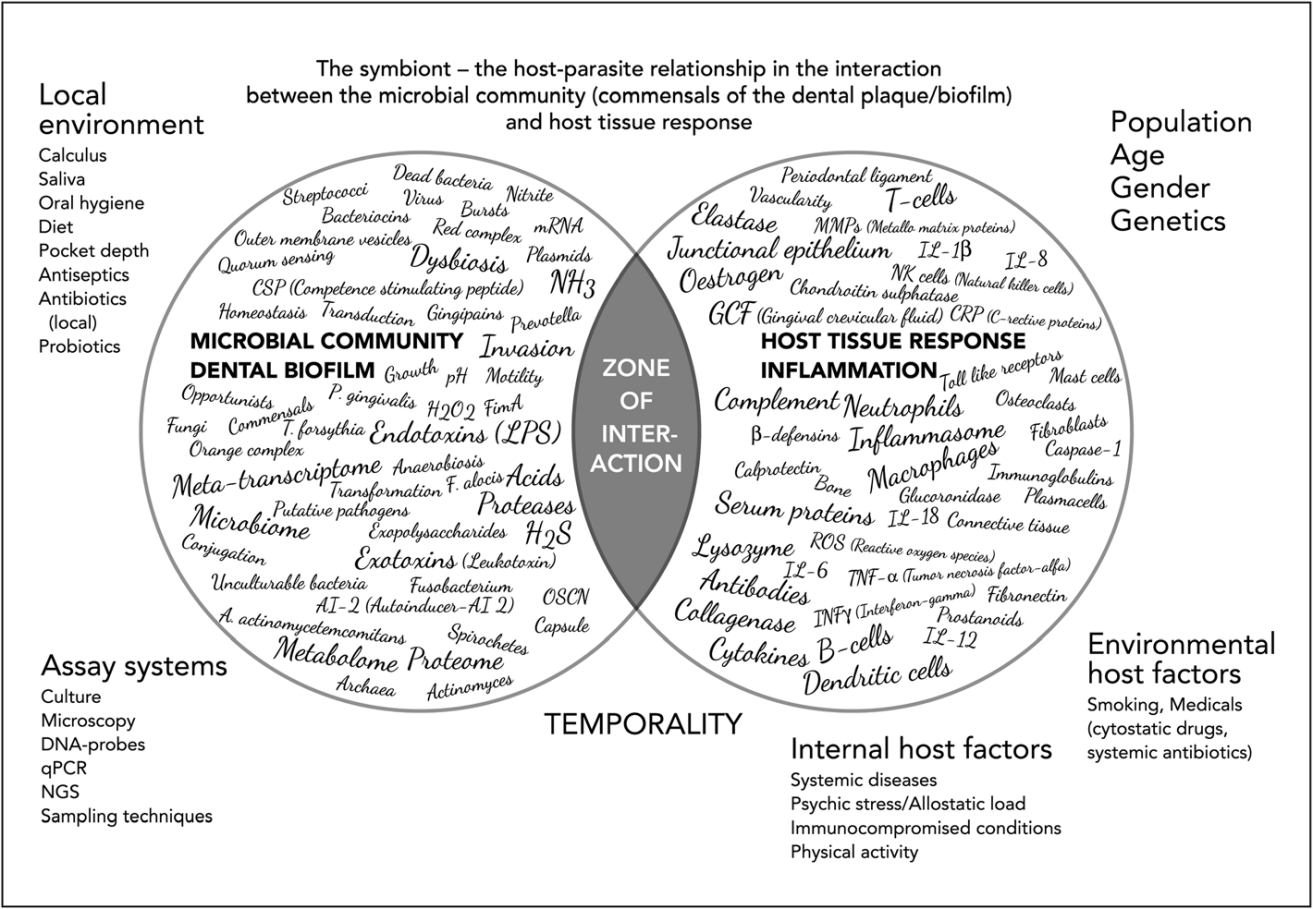
The symbiont-the host-parasite relationship in the interaction between the microbial community within the subgingival dental plaque/biofilm and the host tissue response in inflammation. The factors given within the microbial community represent those that have claimed to be of importance for the activities within the biofilm as well as exposing the host tissues [14, 16, 57]. Similarly, the factors given within the host tissue response are those usually claimed to participate in the inflammatory reaction or as host defence factors against infections. The subgingival microbial community (dysbiosis) is under influence of local environmental factors such as saliva, oral hygiene, diet, pocket depth, antiseptics, antibiotics (local) and probiotics. The composition and activity within the dental biofilm are highly dependent on the assay systems used for evaluation e.g. culture, microscopy DNA-probes, quantitative polymerase chain reaction (qPCR) or next generation sequencing (NGS), biochemical methods and sampling techniques and strategies. The host tissue response of each individual is influenced by population, age, gender and genetics [16]. Environmental host factors such as medicals (cytotoxic drugs, systemic antibiotics) and smoking [17] as well as internal host factor such as systemic diseases and conditions (e.g. diabetes, obesity) [59], psychic stress/allostatic load [60] The two systems are highly dynamic and constantly fluctuating in activity and characterized by temporality. Abbrevations: NH_3_ ammonia, H_2_S hydrogen sulphide, LPS lipopolysaccharide, OSCN- hypothiocyanite, H_2_O_2_ hydrogen peroxide, AI-2 Autoinducer-2, CSP Competence-stimulating peptide, GCF Gingival crevicular fluid, IL interleukins (IL-1beta, IL-6, IL-8, IL.-18), TNFalfa Tumor necrotic factor alfa, IFNgamma Interferon gamma, MMP’s Matrix Metalloproteinases, ROS reactive oxygen species, CRP C-reactive protein

Gingival inflammation may result in a deepening of the gingival pocket as a result of swelling and oedema, all of which favour an increasing flow of GCF, influencing the type of microorganisms that are able to colonize the space [12, 55]. Similarly, the lowering redox potential (Eh) favours the anaerobes. In contrast to the supragingival plaque, adhesion of the microorganisms does not play a crucial role in the gingival pocket and motile bacteria (*Treponema, Campylobacter, Selenomonas* species) are able to establish themselves by mechanical retention [53]. The GCF contains humoral defence factors (antibodies, complement, antimicrobial peptides) as well as inflammatory cells such as neutrophils and monocytes. Bacteria can escape phagocytosis by producing capsules (*P. gingivalis*) or leukotoxins (*Aggregatibacter actinomycetemcomitans*) or simply by being proteolytic, degrading most proteins including humoral antimicrobial factors such as immunoglobulins (IgG), complement factors or antimicrobial host defence peptide [56, 57]. The exudate also contains lysozyme, an enzyme directed towards the peptidoglycan of the bacterial cell wall, which is protected by the outer membrane of Gram-negative microorganisms. The Gram-negatives appear to have a higher survival rate in the inflamed gingival pocket. Inevitably, inflamed gingival pockets contain a microbiota dominated by Gram-negative, anaerobic, proteolytic, and motile bacteria. Numerous studies have been performed using various strategies and molecular biology methods such as qPCR and Next Generation Sequencing (NGS) that have associated a number of culturable and unculturable microorganisms with periodontitis and the environment present in the periodontal pocket but their role in the disease process remains unclear [14, 58]. The complexity of the many factors involved within the biofilm is shown in Fig. 1.

### Host response

The host response to the microbial challenge in periodontal disease is complex and numerous factors are involved [16]. Figure 1 illustrates the many factors at play. At prolonged exposition of the gingiva for the dental biofilm an immune-response phase involving lymphocytes and plasma cells, is activated. They are more slowly recruited and are activated producing and releasing a cascade of mediators (e.g. interleukins and cytokines) characteristic for a more chronic type of inflammation [16, 57]. In reality, the host response to the dental biofilm is balanced against the bacterial challenge and the activity of the bacterial biofilm characterised by the chronic inflammatory phase and due to continuous fluctuations within the bacterial activity in the dental biofilm also includes various degrees of the acute phase. In addition, the host is under constant or changing influence from external and internal factors such as systemic diseases (e.g. diabetes), medications (cytostatic drugs), smoking and psychological factors (stress, allostatic load), which makes the outcome of the inflammatory response at the individual level during many years of bacterial challenge highly unpredictable [59, 60].

### An alternative perspective on the development of periodontitis

Numerous laboratory and clinical studies have provided valuable insights into many of the necessary and sufficient biological conditions under which periodontitis occurs. However, the results of such studies do not explain the variations in the distribution of the disease nor, indeed, the reason for the apparent universality of the features of periodontal breakdown observed in epidemiological studies [33, 42]. This is primarily because the processes involved in periodontitis are highly complex, with spatial and temporal variations in the number and types of determinants, but also in their relative influence over time. The search for a perfect deterministic model — one that relates perfectly all potential determinants — has not been successful because of the complexity of the processes involved in relation to the composition of the biofilm and the capacity of the host to defend itself (Fig. 1). Even if such a model existed, it would be useless because most of the determinants can, at best, be measured only as proxy variables. Even if we had such a model, periodontitis would be unpredictable since the inputs (times, lengths, frequency and type of diet, GCF flow rates, quality and quantity and composition of plaque, and host defence factors) are highly variable and noisy (complex, variable, and like a room full of people talking at the same time where it is impossible to tell who is saying what).

But this very noisiness could well play a crucial role in the process. Hitherto poorly understood phenomena can sometimes be trivially explained by random process theories. The critical test about such theories is whether they are able to explain the epidemiological and clinical features of the disease process [25, 61].

Amongst the many characteristics of periodontitis are that age-related changes appear to be visible only when pronounced loss of attachment is examined, whereas age related changes are much less apparent when the milder forms of attachment loss are considered since milder forms of attachments loss tend to be ubiquitous in adult populations [19]. In addition, the change in the risk of loss of attachment occurring following eruption of a tooth into the mouth is very small, but increases with age in adulthood. At the same time, it is apparent that the longer a tooth surface survives without marked loss of attachment, the less likely is it to occur.

These phenomena are consistent with simplest of random process models — Brownian motion, as described by Einstein [62].

**Fig. 2:**
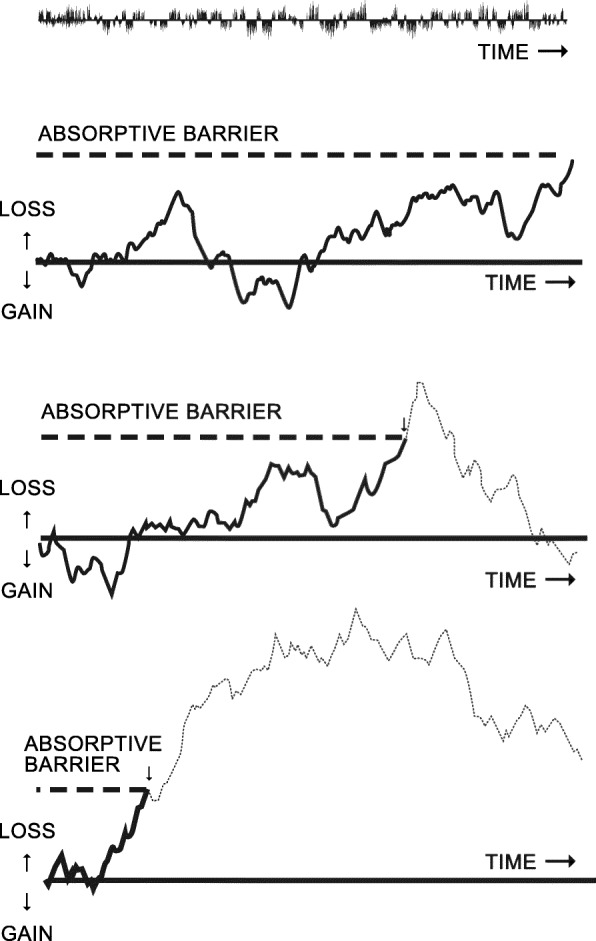
**a-c.** Three examples of a potentially infinite series showing erratic loss and gain of tissues caused by accumulation of (integrating the effects of small random fluctuations of activity (noise) within the dental biofilm. Adopted from Manji et al. [26, 61]

Given how many factors involved, and given how little we know about what may be happening at any given moment, we can reasonably consider the influence of the multitude of determinants involved over a given period as being effectively random and constituting ‘noise’, some enhancing inflammation, some enhancing recovery, and some perhaps having no influence. While there may be noisy events happening within the biofilm, it is necessary to consider how the tissues are affected by this noise: the tissues respond to these stimuli by *accumulating* the effects of all the different components of noise (that is to say, the effects on the tissues of the positive, negative and neutral events are added together). This can illustrate by generating random numbers of positive and negative values, each with an equal probability of occurring (with an average value of zero). By adding together these numbers, we can observe unpredictable variations, sometimes substantial rise in their values and sometimes substantial decline. If we consider the response of the gingival tissues accumulating the noise within the biofilm with which it is in contact, the tissues would experience unpredictable bursts and remissions, inflammation and recovery, despite the noise within the biofilm being at a steady state or homeostatic. We would thus expect that the gingiva becomes inflamed for an unpredictable period, and then recover for an unpredictable period. That is in fact what we can observe in experimental gingivitis studies – the phenomenon of unpredictable inflammation and recovery of the gingivae [63]. The tissue reaction would look like the following set of graphs (Fig. 2a-c). While the underlying noise may be homeostatic (equal probabilities of positive and negative events) the effect of accumulating the noise leads to unexpected bursts and remissions of activity.

If there are conditions that might enhance inflammation, for example, a weakened immune response, reduction in GCF flows, clumsy probing of periodontium, presence of pathogenic microorganisms, then the balance between inflammation and recovery will be altered, and so the probability of inflammation is increased. Similarly, if the biofilm is disturbed regularly by oral hygiene practices, the probability of recovery is increased.

Here we are assuming that we are dealing only with commensal organisms within the biofilm. These bursts and remissions will occur even in the absence of putative pathogens.

If the random activities of the commensal organisms and of the host defence mechanisms are allowed to continue over time, every now and then the cumulative effects will sometimes be unpredictably so severe that a point of no return is reached (which technically in statistical theory is referred to the ‘absorptive barrier’) where the collagen fibres attaching the gingival tissues to the tooth surface are destroyed by the inflammatory process, resulting in loss of attachment. This can happen even if we consider that within the biofilm the probability of inflammation-inducing and recovery-inducing factors are equal. In other words, this behaviour is intrinsic to a process in which the effects of random noise are accumulated. Thus such breakdown resulting in loss of attachment, can occur over time without any change in the composition of the microbiome or in the capacity of the host to exercise recovery. Naturally, any determinants that are likely to increase the probability of inflammation occurring will increase the probability of loss of attachment to occur.

The statistical properties of this simple random effects model with an absorptive barrier are well known [25, 61, 64]. The model’s probability density function has some interesting features: It generates a cumulative probability curve that is similar to the prevalence of loss of attachment at a given site that would be observed in a cross-sectional study of populations with an age range from eruption of the tooth into the mouth to old age. The model predicts that very few surfaces would be affected shortly after eruption, but in the older age groups there would be an almost complete ubiquity of milder forms of attachment loss, and an almost linear relationship with age in severe loss of attachment. In the oldest age-group the rate at which loss of attachment continues may eventually slow down. The rate at which such loss of attachment occurs depends, of course, on the presence of determinants that increase or decrease the probability of inflammatory reactions or capacity of tissue recovery.

Most interesting of all is the model’s hazard function [64], that is, the instantaneous probability of a loss of attachment occurring at a given site which until then has survived without having developed one. The model predicts that loss of attachment in the early period after eruption is highly unlikely. Thereafter, the hazard function reaches a peak and subsequently the longer a surface survives without exhibiting any loss of attachment, the less likely is it that it will occur thereafter.

The model described here is an idealization of the processes that occur in reality and assumes that each of the determinant variables involved in the development of periodontitis are independent. However, any positive correlations of inputs would only enhance the degree of inflammation and recovery of the tissues, whereas negatively correlated events would tend to dampen them. In essence, however, the model holds true in either case. The assumptions made are, therefore, not unreasonable.

The model predicts that if one includes all degrees of loss of attachment, then the degree of change with age is relatively little, whereas with advanced loss of attachment the linear relationship with age is evident. This is a result of the *intrinsic* nature of the process that lead to the loss of attachment. And this may explain why in vastly different populations we see similar patterns of loss of attachment with approximately the same proportion of the older people exhibiting advanced loss of attachment. In practice, the phenomenon of periodontitis being age related may also be influenced by the degree to which aging itself may reduce the capacity of the collagen fibres to recover the ever ongoing inflammatory processes [65].

This simple random effects model predicts many of the features of periodontitis that are observed in epidemiological studies. It does not require one to postulate the role of one or other pathogenic microorganism or any particular determinant in the etiology of the disease, although of course such factors would increase the probability of loss of attachment. On the contrary, one requires only to have commensal microorganisms to result in the pattern of loss of attachment observed in epidemiological studies in many varied populations.

The model gives expression to the concept of periodontitis as a process involving the tissues *accumulating* the effectively random noise of inflammatory provocations and factors promoting recovery within the biofilm in contact with the tissues that over long periods of time result in breakdown of the tissues and loss of attachment. The model predicts the occurrence of bursts and remissions in the progress of periodontitis [25]. The model suggests that some degree of loss of attachment is likely to occur after 30–40 years of age, but that simple measures to disturb the biofilm regularly (oral hygiene) may reduce the probability of loss of attachment.

## Conclusions

The model described here complements other more deterministic theories. Using existing knowledge and insights about the development of periodontitis, and making few assumptions, it provides parsimonious and simple explanations for a number of phenomena that have hitherto proved difficult, or have required complex arguments, to explain. What this model offers is one way in which the noise itself may be considered the subject of interest for enhancing our understanding of periodontitis.

## Data Availability

All data are available through cited references.

## Abbrevations

GCF: Gingival Crevicular Fluid
qPCR: Quantitative Polymerase Chain Reaction
NGS: Next Generation Sequencing
